# Impact of the severe familial hypercholesterolemia status on atherosclerotic risks

**DOI:** 10.1038/s41598-023-47147-z

**Published:** 2023-11-13

**Authors:** Hayato Tada, Atsushi Nohara, Soichiro Usui, Kenji Sakata, Masa-aki Kawashiri, Masayuki Takamura

**Affiliations:** 1 Department of Cardiovascular Medicine, Graduate School of Medical Sciences, Kanazawa University, 13-1 Takara-machi, Kanazawa, 920-8641 Japan; 2 Department of Clinical Genetics, Ishikawa Prefectural Central Hospital, Kanazawa, Japan; 3 Department of Internal Medicine, Kaga Medical Center, Kaga, Japan

**Keywords:** Cardiology, Risk factors

## Abstract

Risks of atherosclerotic events substantially vary even among patients with familial hypercholesterolemia (FH) with extremely high risk based on life-long exposure to high low-density lipoprotein cholesterol levels. This study aimed to examine the impact of the severe FH status defined by the International Atherosclerosis Society (IAS). Data of patients with FH (N = 1050, male = 490) who were admitted to Kanazawa University Hospital between 2000 and 2020 and who were followed up were retrospectively reviewed. The number of major adverse cardiac events (MACEs), including mortality associated with cardiovascular disease, acute coronary syndrome, and ischemic heart disease requiring coronary revascularization per 1000 person-years, was calculated. Hazard ratio was also calculated using Cox proportional model. Overall, 545 (51.9%) patients had severe FH. The median follow-up duration was 12.6 years. In total, 171 MACEs were recorded during the follow-up period. Severe FH was significantly associated with MACE (hazard ratio = 6.48, 95% confidence interval = 2.56–10.40, *P* = 1.2 × 10^−5^). The event rates per 1000 person-years in the primary prevention group of non-severe FH and severe FH, were 0.0 and 15.6, respectively. The event rates per 1000 person-years in the secondary prevention group of non-severe FH and severe FH, were 2.0 and 32.3, respectively. Patients with severe FH exhibited significantly higher risks in primary and secondary prevention settings. This simple criterion provides useful information for identifying patients with even higher risk who may need further management.

## Introduction

Cardiovascular diseases (CVDs) have been the most frequent causes of death worldwide^1^; thus, cardiologists have been trying to risk stratify high-risk patients. In this sense, patients with familial hypercholesterolemia (FH) caused by pathogenic mutations in the low-density lipoprotein receptor (*LDLR*) or its associated genes, including apolipoprotein B (*APOB*), proprotein convertase subtilisin/kexin type 9 (*PCSK9*), and *LDLR* adaptor protein 1 (*LDLRAP1*), are at extremely high risk of CVDs^2–4^. However, CVD risks are considerably different even among these high-risk patients. For example, we have previously shown that classical risk factors such as hypertension, diabetes, and smoking, as well as emerging risk factors, such as remnant cholesterol, lipoprotein (a) [Lp(a)], genetic variants, and subclinical atherosclerosis, were significantly associated with CVD risk even among patients with heterozygous FH (HeFH)^5–9^. Recently, the International Society of Atherosclerosis (IAS) proposed the unique concept of “severe FH” where CVD risk of HeFH appears to increase substantially^10^. Several studies have tried to validate the clinical effect of this status in independent FH cohorts, especially among patients under the primary prevention settings^11,12^. Funabashi et al. showed that a “severe FH” status was significantly associated with CVD events among Japanese patients with FH under the primary prevention settings^13^. However, whether this concept fits into patients under the secondary prevention settings is still unclear. Accordingly, this study aimed to reassess the clinical impact of a “severe FH” status on future CVD events among independent Japanese cohort, including patients under the secondary prevention settings.

## Patients and methods

### Study population

This study analyzed data collected from 2042 patients diagnosed with FH using the Japan Atherosclerosis Society (JAS) 2017 criteria^14,15^. These patients were admitted to Kanazawa University Hospital between 2000 and 2020 and were followed up. Some patients were excluded because of missing data (n = 491), homozygous FH (n = 6), and lost to follow-up (n = 495). Finally, 1050 patients were included in the study (Supplemental Fig. 1).

### Clinical data assessment

Baseline was defined as the point when initial assessments were performed before the initiation of lipid-lowering therapies, and the follow-up was the point when latest assessments were performed. Hypertension was defined as a systolic blood pressure of ≥ 140 mmHg and/or diastolic blood pressure of ≥ 90 mmHg or the use of antihypertensive medications. The definition of diabetes by the Japan Diabetes Society was used^16^. Smoking status was defined as current smoking status. CVD was defined as the presence of myocardial infarction, unstable angina, or coronary artery revascularization. The total serum cholesterol, triglyceride, and high-density lipoprotein (HDL) cholesterol levels were determined enzymatically via automated instrumentation. LDL cholesterol levels were calculated using the Friedewald formula if the triglyceride level was < 400 mg/dL; otherwise, it was determined enzymatically. The LDL cholesterol year score was calculated as LDL cholesterol max × [age at diagnosis/statin initiation] + LDL cholesterol at inclusion × [age at inclusion − age at diagnosis/statin initiation]^17^. The LDL cholesterol levels during LDL apheresis therapy were calculated as follows: C_average_ = C_min_ + 0.73 (C_max_–C_min_), where C_average_ is the mean concentration during biweekly LDL-apheresis therapy and C_max_ and C_min_ are the concentration just before and after a single session of LDL-apheresis^18^. In this study, the LDL cholesterol level 2 weeks after the use of PCSK9 inhibitor was used for patients taking this drug. Major adverse cardiac event (MACE) was defined as mortality associated with CVD, acute coronary syndrome, and ischemic heart disease requiring coronary revascularization. According to IAS, severe FH was LDL cholesterol > 400 mg/dL, LDL cholesterol > 310 mg/dL plus 1 high-risk feature, or LDL cholesterol > 190 mg/dL plus high-risk features. High-risk features included age > 40 years without treatment, smoking, male sex, Lp(a) > 50 mg/dL (75 nmol/L), low-HDL cholesterol (< 1 mmol/L or 40 mg/dL), hypertension, diabetes mellitus, family history of premature CVD in first-degree relatives (age < 55 years in men and < 60 years in women), chronic kidney disease (defined as an estimated glomerular filtration rare < 60 mL/min/1.73 m^2^, and body mass index > 30 kg/m^2^)^10^. High-intensity statins were defined as the maximum approved doses of strong statins on FH in Japan (rosuvastatin 20 mg, atorvastatin 40 mg, or pitavastatin 4 mg).

### Genetic analysis

A next-generation sequencer was used to evaluate genotypes. Briefly, the coding regions of *APOB*, *LDLR*, *LDLRAP1*, and *PCSK9* were sequenced, as described in a previous study^19^. Moreover, copy number variations at *LDLR* were assessed using the eXome Hidden Markov Model, as described previously^20^. The standard American College of Medical Genetics and Genomics criteria (“Pathogenic” or “Likely Pathogenic”) were used to determine whether the genetic variants were pathogenic^21^.

### Ethical considerations

This study was approved by the Ethics Committee of Kanazawa University (2015-219). All procedures met the ethical standards of the Human Research Committee (institutional and national) and the 1975 Declaration of Helsinki (revised in 2008), and Ethical Guidelines for Medical and Health Research Involving Human Subjects, and all other applicable laws and guidelines in Japan. All participants provided informed consent for the genetic analysis.

### Statistical analysis

Continuous variables with normal distribution were presented as means ± standard deviations, whereas continuous variables without a normal distribution were expressed as medians and interquartile ranges (IQRs). All comparisons between categorical variables were performed using Fisher’s exact test or the chi-square test. Data were presented as numbers or percentages. For independent variables, Student’s *t*-test was used to compare the means of continuous variables, and the nonparametric Wilcoxon Mann–Whitney U test was used to compare the median values. The chi-square test or Fisher’s exact test was used to evaluate categorical variables. Multivariable Cox regression hazard model was used to assess MACE-associated factors. The cumulative Kaplan–Meier survival curves were generated to compare the time to the development of the first MACE. For each stratum of the prevention status, the MACE per 1000 person-years was calculated. In addition, we calculated the MACE per 1000 person-years according to the subgroups, including age (50 years or older, sex, and the attainment of LDL cholesterol treatment target where < 100 mg/dL in the primary prevention setting and < 70 mg/dL in the secondary prevention setting). *P*-values of < 0.05 were used to denote statistical significance.

## Results

### Clinical characteristics of the participants

Characteristics of the participants are illustrated in Table 1. The mean age was 49 years, and 47% were men. The median LDL cholesterol and lipoprotein (a) [Lp(a)] levels at baseline were 239 and 20.4 mg/dL, respectively. We found a pathogenic variant as FH among 777 (74.0%) patients. As expected, we found significant differences in variables, including age, sex, diabetes, hypertension, smoking, total cholesterol, HDL cholesterol, baseline LDL cholesterol, LDL cholesterol year score, prevalence of family history of premature CVD, prevalence of tendon xanthomas, and prevalence of prior CVD. When we divided the patients according to sex, we found several differences in several parameters, including LDL cholesterol on treatment (Supplemental Table 1). One hundred seventy one MACEs (Supplemental Table 2) were recorded during the follow-up period (median follow-up duration [interquartile range]: 12.6 [9.1–17.4] years).

**Table 1:** Baseline characteristics of the participants.

Variables	All (N = 1050)	Severe FH (N = 545)	No severe FH (N = 505)	*P*-value
Age (years)	49 ± 16	58 ± 13	38 ± 17	< 2.2 × 10^−16^
Male (%)	490 (46.7%)	349 (64.0%)	141 (27.9%)	< 2.2 × 10^−16^
Hypertension (%)	250 (23.8%)	235 (43.1%)	15 (3.0%)	< 2.2 × 10^−16^
Diabetes (%)	83 (7.9%)	72 (13.2%)	11 (2.2%)	7.7 × 10^−11^
Smoking (%)	301 (28.7%)	256 (56.1%)	45 (8.9%)	< 2.2 × 10^−16^
Total cholesterol (mg/dL)	318 [268–365]	347 [308–398]	301 [267–334]	< 2.2 × 10^−16^
Triglyceride (mg/dL)	113 [76–177]	134 [95–178]	113 [74–153]	5.1 × 10^−5^
HDL cholesterol (mg/dL)	47 [43–51]	44 [37–55]	52 [44–61]	1.3 × 10^−13^
LDL cholesterol (at baseline, mg/dL)	239 [208–279]	276 [222–314]	224 [198–257]	< 2.2 × 10^−16^
Lp(a) (mg/dL)	20.4 [10.6–40.5]	33.4 [15.6–50.1]	15.5 [9.2–30.2]	< 2.2 × 10^−16^
LDL cholesterol year score at baseline (years × mg/dL)	11,806 [8429–15,530]	14,120 [11,411–17,583]	8908 [5696–11,963]	< 2.2 × 10^−16^
FH pathogenic variants (%)	777 (74.0%)	403 (73.9%)	374 (74.1%)	1.0
Family history of premature CVD	288 (27.4%)	209 (38.3%)	79 (15.6%)	3.1 × 10^−16^
Tendon xanthomas	533 (50.8%)	398 (73.0%)	135 (26.7%)	< 2.2 × 10^−16^
prior CVD (%)	295 (28.1%)	256 (47.0%)	39 (7.7%)	< 2.2 × 10^−16^

CVD, cardiovascular disease; FH, familial hypercholesterolemia; MACE, major adverse cardiac event.

### LDL cholesterol year score according to the severe FH status

**Figure 1:**
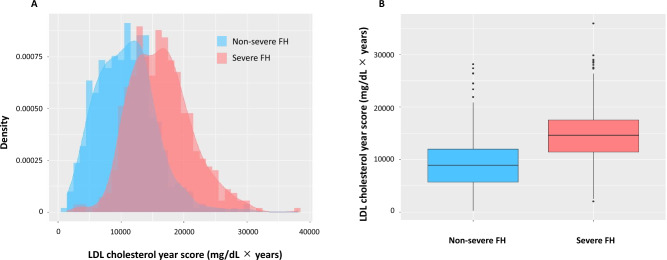
LDL cholesterol year score according to the incidence of MACEs. (**A**) Histograms with density: Red and green indicate patients without and with MACEs, respectively. (**B**) Boxplots: Red and green indicate patients without and with MACEs, respectively.

The LDL cholesterol year score at follow-up, which represented LDL cholesterol accumulation in patients with and without severe FH, had a normal distribution (Fig. 1A). At baseline, the median LDL cholesterol year score in the severe FH group was significantly higher than that in the non-severe FH group (14,120 [11,411–17,583] vs. 8908 [5696–11,963] mg/dL, *p* < 2.2 × 10^–16^, respectively) (Fig. 1B).

### Medical therapies according to the severe FH status

Table 2 shows the follow-up medical treatments. Most patients received statins, followed by ezetimibe, and colestimide. The proportions of these combination therapies were higher in the severe FH group than in the non-severe FH group. In addition, we found that the proportion of the patients treated with high-intensity statins were significantly higher in severe FH patients. However, during treatment, the LDL cholesterol level of the severe FH group was still higher than that of the non-severe FH group.

**Table 2:** Medical therapies.

	All (N = 1050)	Severe FH (N = 545)	No severe FH (N = 505)	*P*-value
LDL cholesterol at baseline (mg/dL)	239 [208–279]	276 [222–314]	224 [198–257]	< 2.2 × 10^−16^
Statins (%)	1025 (97.6%)	536 (98.3%)	489 (96.8%)	0.16
High-intensity statins (%)	278 (26.4%)	188 (34.5%)	90 (17.8%)	1.5 × 10^−9^
Ezetimibe (%)	644 (61.3%)	380 (69.7%)	264 (52.3%)	9.6 × 10^−9^
Colestimide (%)	243 (23.1%)	125 (22.9%)	118 (23.4%)	0.93
Probcol (%)	2 (0.2%)	2 (0.4%)	0 (0.0%)	0.51
PCSK9 inhibitor (%)	45 (4.3%)	32 (5.9%)	13 (2.6%)	0.01
LDL apheresis (%)	2 (0.2%)	2 (0.4%)	0 (0.0%)	0.51
Fibrates (%)	6 (0.6%)	3 (0.6%)	3 (0.6%)	1.0
n-3 PUFAs (%)	10 (1.0%)	7 (1.3%)	3 (0.6%)	0.41
LDL cholesterol on treatment (mg/dL)	112 [96–120]	117 [101–131]	104 [90–118]	0.003

PCSK9, proprotein convertase subtilisin/kexin type 9; PUFA, polyunsaturated fatty acids.

### MACE-associated factors

In this study, age (hazard ratio [HR] = 1.05, 95% confidence interval [CI] = 1.02–1.08, *p* = 3.2 × 10^–7^, Table 3), male sex (HR = 1.50, 95% CI = 1.04–1.96, *p* = 0.02), hypertension (HR = 2.10, 95% CI = 1.44–2.76, *p* = 2.4 × 10^−5^), diabetes (HR = 1.66, 95% CI = 1.10–2.22, *p* = 0.001), smoking (HR = 2.56, 95% CI = 1.76–3.36, *p* = 0.0001), LDL cholesterol at baseline (HR = 1.01, 95% CI = 1.00–1.02, *p* = 0.03, per 10 mg/dL), pathogenic variants (HR = 2.46, 95% CI = 1.54–3.38, *p* = 6.9 × 10^–5^), and prior CVD (HR = 3.56, 95% CI = 2.24–4.88, *p* = 4.2 × 10^–6^) were associated with MACE. Under the circumstances, a severe FH status was significantly associated with MACE (HR = 6.48, 95% CI = 2.56–10.40, *p* = 1.2 × 10^–5^).

**Table 3:** Factors associated with MACE.

Variable	HR	95% CI	*P*-value
Age (per year)	1.05	1.02–1.08	3.2 × 10^−7^
Male (yes vs. no)	1.50	1.04–1.96	0.02
Hypertension (yes vs. no)	2.10	1.44–2.76	2.4 × 10^−5^
Diabetes (yes vs. no)	1.66	1.10–2.22	0.001
Smoking (yes vs. no)	2.56	1.76–3.36	0.0001
LDL cholesterol (per 10 mg/dL)	1.01	1.00–1.02	0.03
pathogenic variants (vs. without variants)	2.46	1.54–3.38	6.9 × 10^−5^
prior CVD	3.56	2.24–4.88	4.2 × 10^−6^
Severe FH	6.48	2.56–10.40	1.2 × 10^−5^

CVD, cardiovascular disease; FH, familial hypercholesterolemia; MACE, major adverse cardiac event.

### Prognosis according to the severe FH status

**Figure 2:**
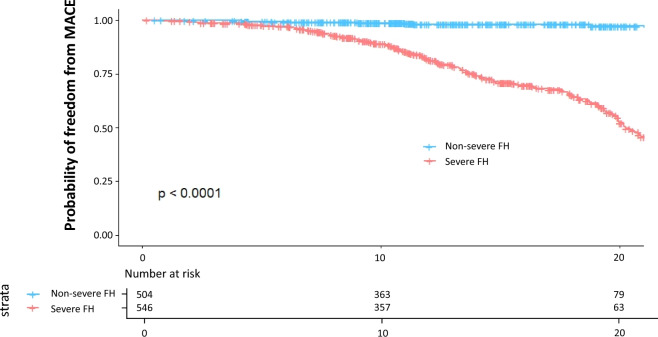
Kaplan–Meier survival curves according to the severe FH status. Blue and red indicate patients with non-severe and severe FH.

**Figure 3:**
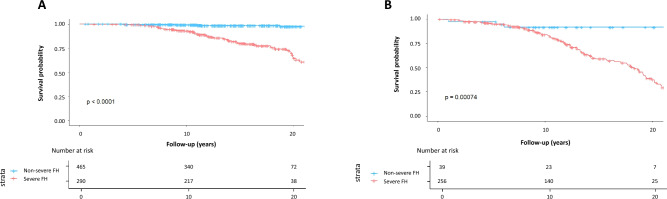
Kaplan–Meier survival curves according to the severe FH status. Blue and red indicate patients with non-severe and severe FH, respectively. (**A**) Primary prevention group. (**B**) Secondary prevention group.

**Figure 4:**
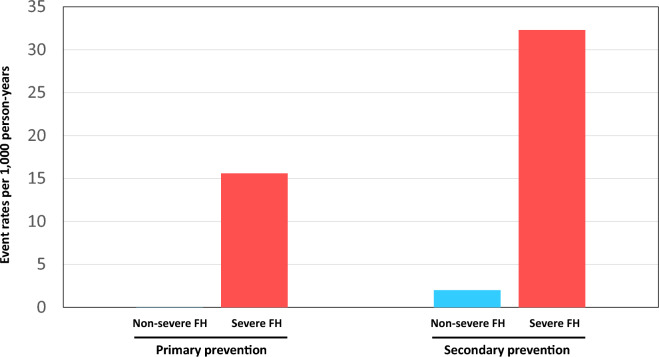
Event rates per 1000 person-years. Blue and red indicate patients with non-severe and with severe FH, respectively.

**Figure 5:**
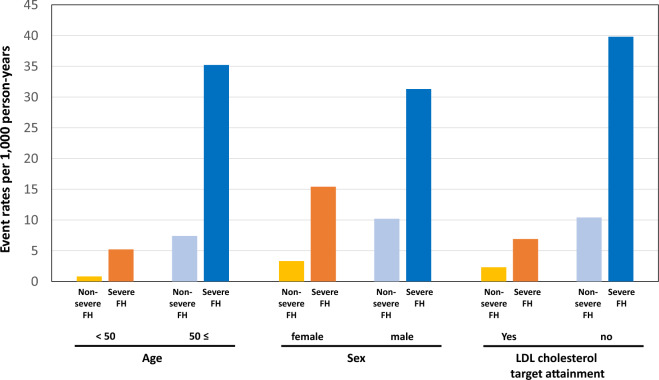
Event rates per 1000 person-years among subgroups of severe FH patients. Orange indicate patients with severe FH and young (< 50 years) or female, or those who attained LDL cholesterol treatment target. Yellow indicate patients with non-severe FH and young (< 50 years) or female, or those who attained LDL cholesterol treatment target. Blue indicate patients with severe FH and old (≥ 50 years), or male, or those who did not attain LDL cholesterol treatment target. Light blue indicate patients with non-severe FH and old (≥ 50 years), or male, or those who did not attain LDL cholesterol treatment target.

Overall, the severe FH group showed significantly worse prognosis than the non-severe FH group (Fig. 2). When the patients were divided into two groups according to the prevention status (primary or secondary), the severe FH group showed significantly worse prognosis than the non-severe FH group in both primary and secondary prevention status settings (Fig. 3). The event rates per 1,000 person-years in the primary prevention group of the non-severe FH and severe FH were 0.0 and 15.6, respectively. The event rates per 1000 person-years in the secondary prevention group of the non-severe FH and severe FH were 2.0 and 32.3, respectively (Fig. 4). The event rates in the subgroups, including age (50 years or older, sex, and the attainment of LDL cholesterol treatment target where < 100 mg/dL in the primary prevention setting and < 70 mg/dL in the secondary prevention setting) among severe FH patients were illustrated in Fig. 5. We found that the event rate of younger group of non-severe FH and severe FH group were 0.8 and 5.2, respectively, and the event rate of older group of non-severe FH and severe FH group were 7.4 and 35.2, respectively. And the event rate of female of non-severe FH and severe FH group were 3.3 and 15.4, respectively, and the event rate of male group of non-severe FH and severe FH group were 10.2 and 31.3, respectively. And the event rate of patients who attained LDL cholesterol treatment target of non-severe FH and severe FH group were 2.3 and 6.9, respectively, and the event rate of the patients who did not of non-severe FH and severe FH group were 10.4 and 39.8, respectively.

## Discussion

This study investigated the clinical impact of a “severe FH” status on future CVD events in an independent Japanese cohort, including patients under the secondary prevention setting. In this study, a severe FH status was significantly associated with MACEs both in the primary and secondary prevention settings. In fact, we found that the prognosis of the non-severe FH group was quite good.

The most important aspect of this disease is early diagnosis because earlier diagnosis and starting treatments lead to their better prognosis^22,23^. Thus, JAS complied and updated the clinical guidelines of FH for adults (aged ≥ 15 years) and children (aged ≤ 15 years), stipulating the diagnostic criteria and LDL cholesterol treatment targets^24,25^. We anticipate that the diagnostic rate of FH will increase because the new version of the clinical criteria of FH accepts genetic testing of FH, which is now covered by the Japanese national health insurance. However, uniform treatment target goals of LDL cholesterol for all patients with this disease are sometimes inadequate to prevent cardiovascular events. Numerous clinical situations such as hypertension, diabetes, and smoking; other biomarkers, such as triglycerides, remnant cholesterol, and Lp(a); genetic factors other than FH-associated genes; and subclinical atherosclerosis have been associated with further increased risk among patients with FH^5–9^. For further risk discriminations among patients with FH, several scores, or classifications such as the Montreal-FH-SCORE, SAFEHEART-RE, and so-called severe FH have been established^10,26,27^. Among them, a “severe FH” status appears to work quite well to identify patients with super high risk. Form the viewpoint of precision medicine, patients at super high risk must be identified, and LDL-cholesterol lowering together with interventions for modifiable additional risk factors must be intensified. In this study, we validated that patients with severe FH under the primary prevention setting had significantly higher risk for MACE than those with non-FH, and that the same is true in patients under the secondary prevention setting. Accordingly, we need to reshape our treatment strategy for patients with FH according to the severe FH status. In addition, we found the gender-gap in LDL cholesterol treatment where LDL cholesterol level in female was significantly higher than that of male. Moreover, we found that the use of high-intensity statins among patients with FH, including severe FH was inadequate. Importantly, we found that the severe patients who attained LDL cholesterol treatment target had better prognosis compared with those who did not. Accordingly, we need to intensify LDL cholesterol treatment in severe FH patients who did not attain LDL cholesterol treatment target.

This study had several limitations. First, this was a single-center retrospective study, and thus our findings might not be applicable to other patients. However, we believe that this is the largest study at least in Japan with a reasonable sample size. Second, we could not account for changes in treatments, including LDL-lowering therapies in detail during the study period. Third, some patients were excluded from the analysis because of missing data or lost to follow-up, which could have affected the results.

## Conclusions

Patients with severe FH exhibited significant higher risks in the primary and secondary prevention settings. This simple criterion provides useful information to identify patients who have even higher risk and may need further managements.

### Supplementary Information

Supplementary Information.

## Data Availability

The datasets used in the current study available from the corresponding author on reasonable request.
