# Supplementary material for: Impact of the severe familial hypercholesterolemia status on atherosclerotic risks
Source: Sci Rep. 2023 Nov 13;13:19782. doi: 10.1038/s41598-023-47147-z (PMC10643630; doi:10.1038/s41598-023-47147-z)
Supplement: Supplementary file 1 — Supplementary Information. [file 41598_2023_47147_MOESM1_ESM.docx]

**Supplemental Material**

**Impact of the Severe Familial Hypercholesterolemia Status on Atherosclerotic Risks**

Hayato Tada^a^* MD, Akihiro Nomura^a^ MD, Atsushi Nohara^b^ MD, Soichiro Usui^a^ MD, Kenji Sakata^a^ MD, Kenshi Hayashi^a^ MD, Noboru Fujino^a^ MD, Masa-aki Kawashiri^c^ MD, Masayuki Takamura^a^ MD.

^a^Department of Cardiovascular Medicine, Graduate School of Medical Sciences, Kanazawa University, Kanazawa, Japan

^b^Department of Clinical Genetics, Ishikawa Prefectural Central Hospital, Kanazawa, Japan

^c^Department of Internal Medicine, Kaga Medical Center, Kaga, Japan

**Supplemental Figure 1. STROBE flow diagram for study inclusion**………………….…2

**Supplemental Table 1. Characteristics according to sex**……...…………..………………3

**Supplemental Table 2. Type of MACEs**……...……………..……………………………...4

**Supplemental Figure 1. STROBE flow diagram for study inclusion**

**
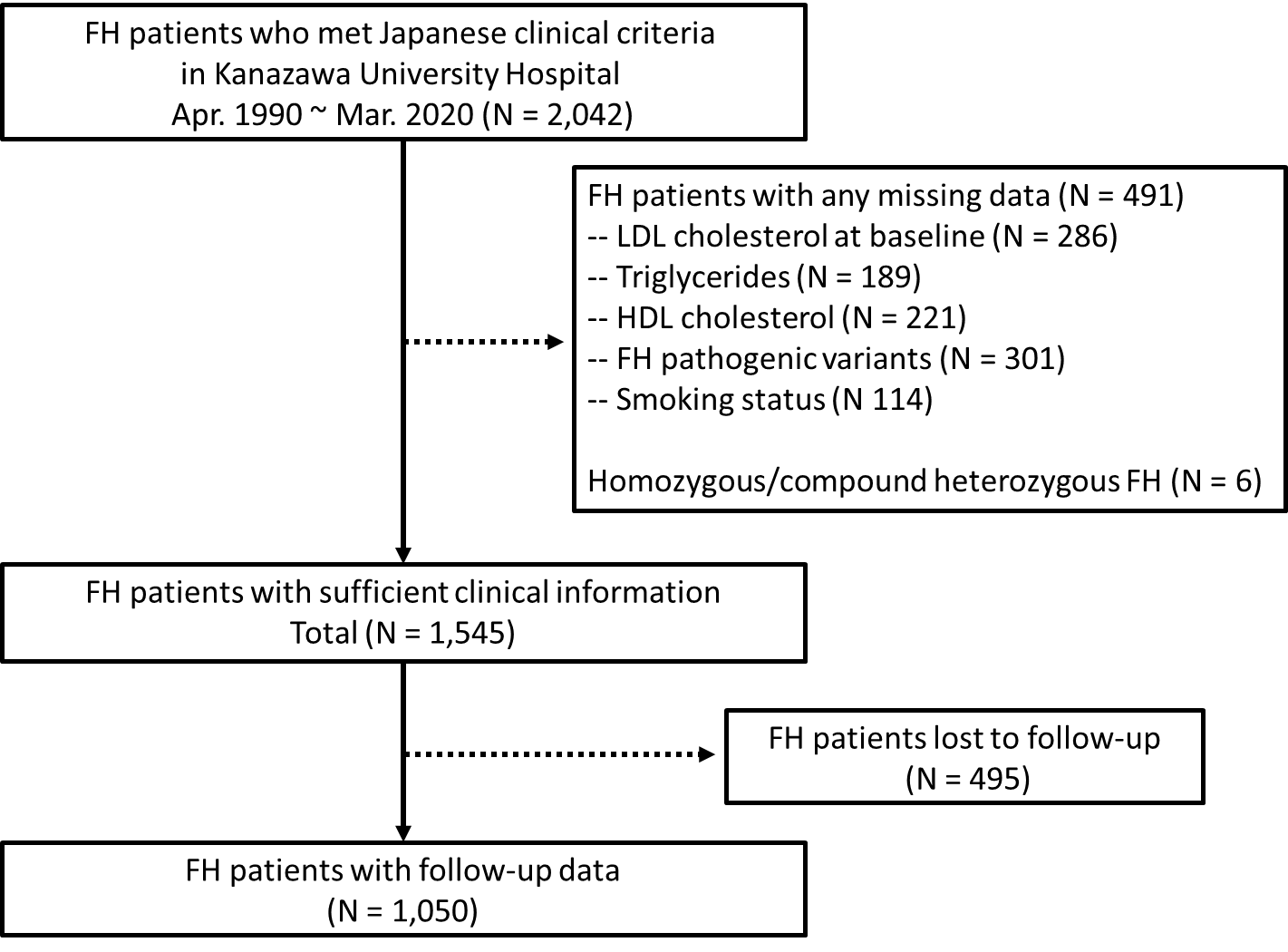
**

**Supplemental Table 1. Characteristics according to sex**

| Variables | All | Male | Female | *P*-value |
| --- | --- | --- | --- | --- |
|  | (N = 1,050) | (N = 490) | (N = 560) |  |
| Age (years) | 49 ± 16 | 44 ± 15 | 56 ± 17 | <2.2 × 10^−16^ |
| Hypertension (%) | 250 (23.8%) | 116 (23.7%) | 134 (23.9%) | 1 |
| Diabetes (%) | 83 (7.9%) | 39 (8.0%) | 44 (7.9%) | 1 |
| Smoking (%) | 301 (28.7%) | 217 (44.3%) | 84 (15.0%) | <2.2 × 10^−16^ |
| Total cholesterol (mg/dL) | 318 [268–365] | 316 [266–358] | 320 [270–368] | 0.21 |
| Triglyceride (mg/dL) | 113 [76–177] | 118 [78–178] | 111 [74–170] | 0.18 |
| HDL cholesterol (mg/dL) | 47 [43–51] | 44 [36–50] | 50 [46–58] | 0.021 |
| LDL cholesterol (at baseline, mg/dL) | 239 [208–279] | 236 [206–276] | 241 [210–281] | 0.16 |
| LDL cholesterol (on treatment, mg/dL) | 112 [96–120] | 101 [90–112] | 122 [98–129] | 0.02 |
| Lp(a) (mg/dL) | 20.4 [ 10.6–40.5] | 18.9 [ 10.2–39.6] | 22.7 [11.6–44.6] | 0.09 |
| LDL cholesterol year score at baseline (years × mg/dL) | 11,806 [8,429–15,530] | 10,480 [7,988–14,996] | 12,558 [9,244–16,363] | 0.001 |
| FH pathogenic variants (%) | 777 (74.0%) | 367 (74.9%) | 410 (73.2%) | 0.58 |
| Family history of premature CVD | 288 (27.4%) | 120 (24.5%) | 168 (30.0%) | 0.05 |
| Tendon xanthomas | 533 (50.8%) | 228 (46.5%) | 305 (54.4%) | 0.01 |
| prior CVD (%) | 295 (28.1%) | 174 (35.5%) | 121 (21.6%) | 8.1 × 10^-7^ |

**Supplemental Table 1. Type of MACEs**

| Type of MACE | All (N = 1,050) |  |
| --- | --- | --- |
|  |  |  |
| CVD-associated mortality | 52 (5.0 %) |  |
| Myocardial infarction | 29 (2.8 %) |  |
| Unstable angina | 51 (4.9 %) |  |
| Ischemic heart disease requiring revascularization | 39 (3.7%) |  |
| Total | 171 (16.3 %) |  |

CVD, cardiovascular disease; MACEs, major adverse cardiovascular events
